# Oral Rehabilitation of Parkinson's Disease Patient: A Review and Case Report

**DOI:** 10.1155/2014/432475

**Published:** 2014-01-16

**Authors:** Fouad A. Al-Omari, Mohammed M. Al Moaleem, Sulaiman S. Al-Qahtani, Abdullah S. Al Garni, Syed Sadatullah, Master Luqman

**Affiliations:** ^1^Department of Maxillofacial Surgery and Diagnostic Sciences, College of Dentistry, King Khalid University, P.O. Box 3263, Abha 61471, Saudi Arabia; ^2^Department of Prosthetic Dental Sciences, College of Dentistry, Jazan University, P.O. Box 114, Jazan, Saudi Arabia; ^3^Division of Oral Medicine, Department of Maxillofacial Surgery and Diagnostic Sciences, College of Dentistry, Najran University, Najran, Saudi Arabia; ^4^Division of Periodontology, Department of Preventive Dental Sciences, College of Dentistry, King Khalid University, P.O. Box 3263, Abha 61471, Saudi Arabia; ^5^Division of Oral Biology, Department of Maxillofacial Surgery and Diagnostic Sciences, College of Dentistry, King Khalid University, P.O. Box 3263, Abha 61471, Saudi Arabia; ^6^Division of Oral Pathology, Department of Maxillofacial Surgery and Diagnostic Sciences, College of Dentistry, King Khalid University, P.O. Box 3263, Abha 61471, Saudi Arabia

## Abstract

Parkinson's disease is usually seen in adults in their middle and late ages. Most people with this disease are less likely to opt for dental treatments unless it is an acute condition. Tremors caused by Parkinson's disease can make dental appointments, especially prolonged treatments, a challenge. The case presented here was successfully treated with an immediate denture for the partially edentulous maxillary and mandibular arches. Early morning brief appointments were given for the procedure. Patient was instructed to take the prescribed parkinsonism medications 60 to 90 minutes before the appointment to utilize the advantage of its peak response. Sympathetic and caring approach towards the patient was employed to reduce his anxiety during the procedures. Some modification of technics and materials was adopted to suit the special situation.

## 1. Introduction 

Parkinson's disease is seen in adults in their late middle or old age. The affected patients have uncontrolled movements of the body along with stiffness of muscles [1]. This is due to the progressive degeneration of nerve cells in the brain resulting in a decrease in dopamine levels. Dopamine is a chemical that helps in transmitting messages between cells. Most Parkinson's patients are old and are less likely to opt for dental treatments except for emergencies. Medication used to treat parkinsonism causes xerostomia [2]. This increases the risk of caries and fungal infections. Removable partial dentures tend to be dislodged or swallowed while complete dentures fall out more often. They can even break following sudden jerky movements. If the person is a complete denture wearer then it becomes difficult to wear them, as the disease gets worse. Tremors caused by Parkinson's disease can make dental appointments a challenge. These patients have a hard time opening their mouth for longer time. Anxiety increases the Parkinson's symptoms. It is important that patient should remain calm during dental treatment. It is essential to make the environment stress-free and as serene as possible. Here is one of such cases of a partially edentulous Parkinson's patient successfully treated with an immediate denture.

## 2. Case Report

**Figure 1:**
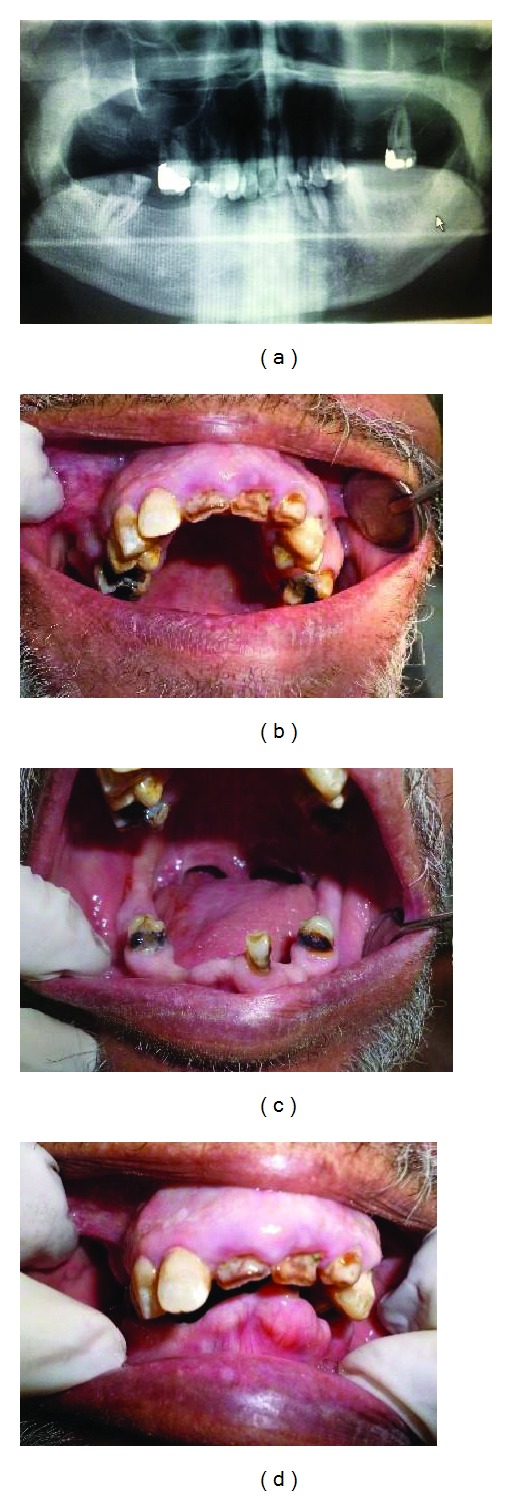
Preoperative status of patient: (a) panoramic view, (b) maxillary arch, (c) mandibular arch, and (d) both arches.

A 62-year-old Saudi male patient reported to King Fahed General Hospital, Jeddah, with complaint of difficulty in eating food due to an ill-fitting partial denture. The patient was looking for complete oral heath check followed by full mouth rehabilitation. Medical history of the patient revealed he was suffering from Parkinson's disease since 18 years with osteoporosis and fracture of the neck and right femur. Signs and symptoms observed included difficulty to stand and walk and stiffness of the joint and muscles with limited movements. He complained of difficulty in brushing teeth, drooling of saliva, and difficulty of speech and swallowing. The patient was undergoing treatment at Department of Neurology in the same hospital. Past dental history included fillings, extraction of teeth, and a removable partial prosthesis. He was able to communicate even though with some difficulty. He was well oriented, did not show signs of depression, and was highly motivated to replace his missing teeth. The intraoral examination (Figures 1(b), 1(c), and 1(d)) revealed dryness of the mouth, instability of the old denture, generalized gingival hyperplasia, and inflammation. While the panoramic radiograph (Figure 1(a)) and hard tissue examination showed dental caries classes III and IV of teeth 13 and 23 (FDI), class II for teeth 14 and 24 (FDI) and remaining roots of teeth 16, 11, 12, 22, 33 and 34 (FDI-Fédération Dentaire Internationale).

**Figure 2:**
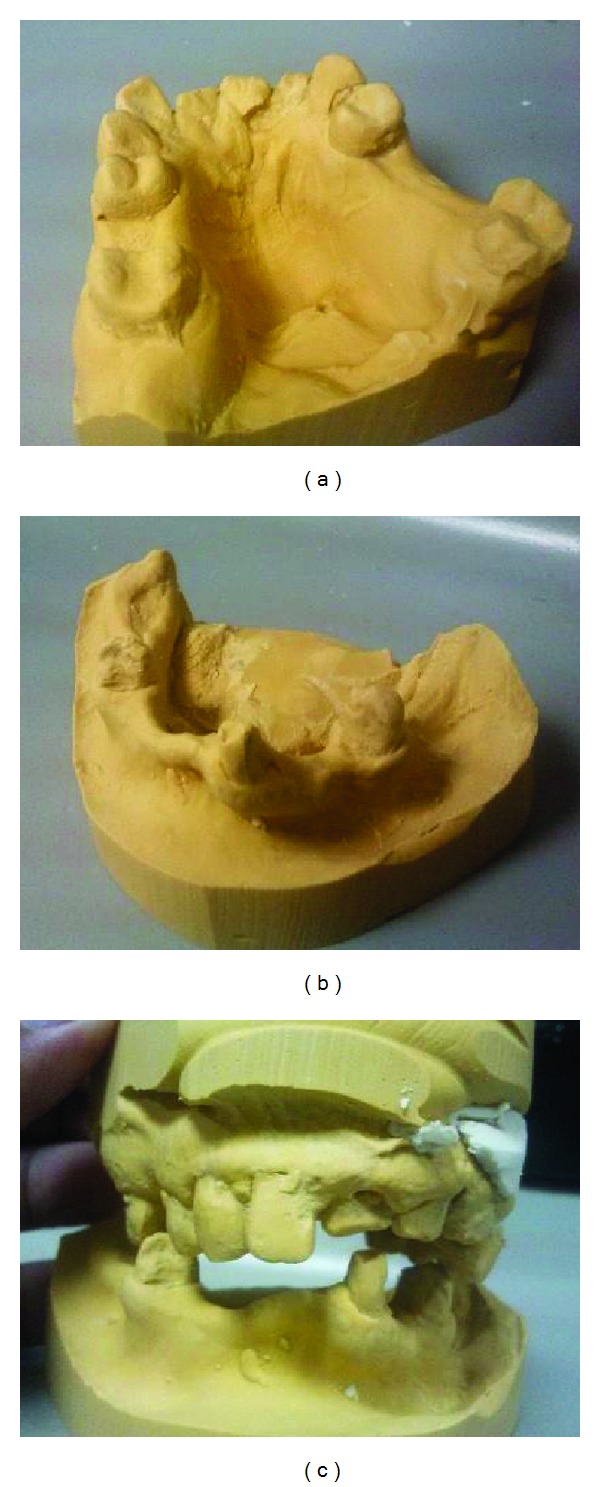
Study cast: (a) maxillary cast, (b) mandibular cast, and (c) both arches together.

After oral examination and radiographic investigation the patient's maxillary and mandibular primary impressions were made with dust-free, fast setting alginate impression material (Jeltrate, Caulk/Dentsply). In the first visit scaling, root planning and polishing were performed and oral hygiene instructions were given to the patient. Care was taken to make the patient comfortable in the operating chair and each step of the procedure was explained in a cordial manner. The study casts were prepared (Figures 2(a), 2(b), and 2(c)) from the alginate impressions for construction of special tray. The next appointment was scheduled early in the morning. It started with multiple restorations with composite resin (Tertic-N-Ceramic, IvoclarVivadent, Lichtenstein) for the decayed teeth. Subsequently maxillary and mandibular final impression with rubber base impression material (Virtual, IvoclarVivadent, Lichtenstein) was made using double mixing technique. This appointment was planned to be short to minimize the patients' mouth opening time. All along the procedure the patient was reassured and consulted regularly for any discomfort.

At the laboratory, mounting of the maxillary and mandibular master cast was done with record block. Slight reduction in the vertical dimension was done to accommodate the interrupting jerky movements of the mandible. Monoplane artificial acrylic teeth were used for arrangement. The dentures were fabricated from high impact strength denture resin Trubyte (Dentsply/Yourk Division). All the laboratory procedures were carried out using a semiadjustable Whip-Mix articulator (WaterpikTechnologies, Fort Collins, CO, USA) after face bow transfer and teeth shade selection. After acrylization the processed immediate dentures were ready for insertion.

**Figure 3:**
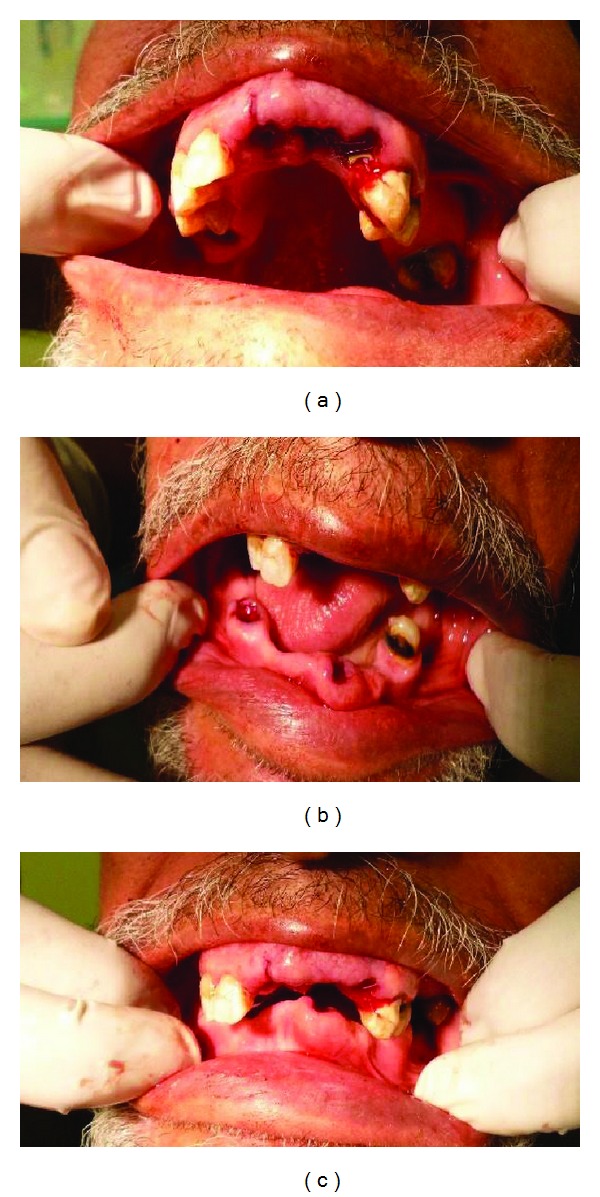
Extraction sites of the remaining roots: (a) maxillary arch, (b) mandibular arch, and (c) both arches together.

**Figure 4:**
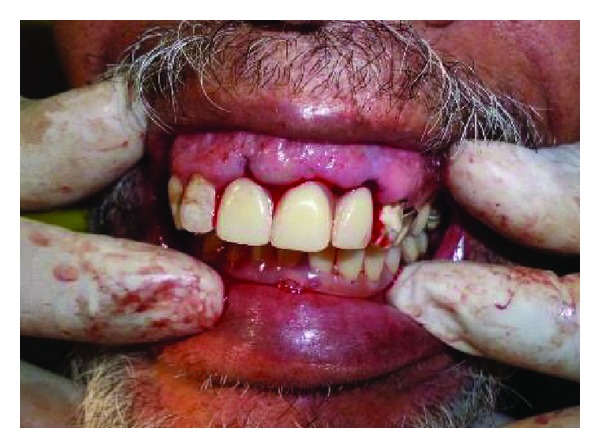
After insertion of maxillary and mandibular immediate dentures.

In the following appointment which was again scheduled in the morning, extraction of the remaining roots for teeth 16, 11, 21, 22, 33 and 34 (FDI) were done (Figures 3(a), 3(b), 3(c), and 3(d)). Following extractions, the maxillary and mandibular immediate dentures were inserted (Figure 4). All the procedures were done with the patient's family member next to the operating chair. The postinsertion instructions were clearly explained to the patient and the family member accompanying him. Follow-up program was scheduled after 2 days, one week, one month, and 3 months intervals for evaluation of the prosthesis.

## 3. Discussions

Symptoms of Parkinson's disease (PD) are a result of insufficient formation and action of dopamine produced in dopaminergic neurons of mid brain [3]. It is accompanied by various signs and symptoms, which affect the day-to-day activities of the patient. The physical symptoms of PD present challenges for daily routine including dental care. Major component of oral hygiene and home care program requires muscle-eye coordination, digital dexterity, and tongue cheek-lip control [4]. Tremor and the associated loss and/or reduction of the above faculties mitigate against effective oral hygiene procedures. Because of the poor motor function, nearly half of all people with PD have difficulty with their daily oral hygiene regimen. For example, they are less likely than others in their age group to clean their dentures daily. Associated problems like rigidity and abnormal posture may make dental examination more difficult. Weakened swallowing ability can increase the risk of aspiration [5]. Additionally, people with PD who have been on medication like levodopa for several years begin to develop dyskinesias, which affects the jaw as well as teeth grinding both of which may create problems during dental treatment [6]. People with PD also experience dry mouth, which contributes to or worsens already existing chewing difficulties or denture discomfort.

In addition to motor-related difficulties associated with PD, there are additional behavioral changes that negatively affect dental care. These include apathy, depression, and forgetfulness, all of which lead to negligence in daily oral health care [7]. PD patients require greater caloric intake than those without PD, but some individuals will actually experience decreased appetite. This problem is worsened with the inability to chew food for denture wearers.

PD patients also experience some level of cognitive impairment, ranging from mild to severe [7]. This may lead to a decline in the practice and effectiveness of many daily self-care routines, including dental hygiene. People who experience cognitive changes may also be more likely to miss dental appointments and less likely to report dental pain to their caregivers or dentist and hence may go unaddressed for too long, further complicating the treatment. The sooner the attention is given to preventive dental care, the better it is for PD patients. Another strategy advocated for PD patients is “one-handed preventive strategies,” which allows them to use the stronger side of the body more often [8].

It is wise to plan for early morning visit, when the waiting time tends to be shorter. It is best to take medication 60 to 90 minutes prior to the office visit to take advantage of a peak response period, which may improve the patient's ability to meet the demands of a dental examination and to avoid the overstress for the patient [2, 9].

Finally, it may be helpful to plan a series of brief office visits rather than few long visits. As PD progresses, the amount of time during which a person responds optimally to their medications will become less, so shorter visit may be more realistic and more productive [9].

Also, as PD progresses, motor symptoms worsen and anxiety may increase, making home dental care and routine dental work more difficult. A neurologist will often be able to help in such situations, weighing the risks of medication with the potential benefit of a dental intervention [9].

The motivation of the patient played a major part for the treatment prognosis in this case. Importance of the previous removable partial denture will give a positive indication for the motivation level of the patient. As the neuromuscular orientation caused interrupted jerky movements of the mandible, the use of monoplane artificial acrylic teeth and reduction in the vertical dimension of the dentures proved beneficial [10]. Caregiver was present with the patient during all dental treatments especially during extraction of teeth [2]. Since Parkinson's patients can develop temporomandibular joint problems combined with severe bruxism, monoplane artificial teeth were chosen for the dentures. In addition, all the restorations were finished with flat occlusal morphology [2, 9]. The patient was instructed to keep the denture outside at night to avoid muscle rigidity of the face associated with acrylic material that is used in the fabrication of this denture [11]. Replacing the missing teeth without changing the vertical dimension was considered the most significant aspect of treating this patient.

## 4. Conclusion

The psychological and behavioral pattern associated Parkinson's disease can cause major difficulties during the fabrication of a dental prosthesis. The success of the prosthesis will depend on the careful approach with diligent handling of the patient during the entire therapy. Educating the patient and the family regarding the post insertion care of the prosthesis is essential for the long term success of the treatment.
